# Supplementary material for: A needs analysis of ESP courses in colleges of art and design: Consensus and divergence
Source: PLoS One. 2024 Jun 11;19(6):e0305210. doi: 10.1371/journal.pone.0305210 (PMC11166353; doi:10.1371/journal.pone.0305210)
Supplement: S1 File — (DOC) [file pone.0305210.s001.doc]

S1 File

**Questionnaire for ESP Needs Survey**

Thank you for taking the time to participate in our survey. Your feedback is invaluable and will greatly contribute to our understanding of art and design education. The survey is to explore and identify the specific needs of English for Specific Purposes (ESP) courses in colleges of art and design. The survey is anonymous and you can choose to terminate it at any time without negative consequences; but if you finish and submit it, the investigators will understand it as a formal consent for using the data in future publications of the research. 感谢您花时间参加我们的调查。您的反馈非常宝贵，将极大地帮助我们了解艺术与设计学院的教学。这项调查旨在探索和识别艺术与设计学院英语专门用途（ESP）课程的具体需求。本调查是匿名的，您可以随时选择终止调查，不会有任何负面后果；但如果您完成并提交调查，调查人员将视为您正式同意在未来的研究出版物中使用这些数据。

For statements 2-23, you will be asked to indicate your level of agreement using a five-point scale. The options on this scale are: 对于第2至23项陈述，您将被要求使用五级量表来表明您的同意程度。这个量表的选项包括：

5 - Strongly Agree: You completely agree with the statement. 5 - 非常同意：您完全同意该陈述。

4 - Agree: You generally agree with the statement, but there might be some exceptions or minor points of disagreement.4 - 同意：您总体同意该陈述，但可能有一些例外或一些小的不同意点。

3 - Neutral: You neither agree nor disagree with the statement, or you might be unsure.3 - 中立/不确定：您既不同意也不反对该陈述，或者您可能不确定。

2 - Disagree: You generally disagree with the statement, although you might find some minor aspects agreeable. 2 - 不同意：您总体不同意该陈述，尽管您可能会发现一些小方面是可以接受的。

1 - Strongly Disagree: You completely disagree with the statement.1 - 非常不同意：您完全不同意该陈述。

Please respond to each statement based on your honest opinions and experiences. There are no right or wrong answers, and your responses will be kept confidential.Thank you again for your participation and valuable insights. 请根据您的真实意见和经验回答每个陈述。没有正确或错误的答案，您的回答将被保密。

1. Please choose your identity.请选择您的身份。

Undergraduate freshmen本科新生

Graduate students 研究生

Art teachers 美术专业教师

English teachers大学英语教师

1. To enhance professional competence or meet work requirements, it is necessary to read English works, literature, and articles in the field of art and design. 为提升专业能力或工作需要，需要阅读本专业的英文作品、文献和文章。
2. To enhance professional competence or meet work requirements, it is necessary to attend courses and lectures delivered by foreign scholars and experts. 为提升专业能力或工作需要，需要听国外学者专家的课程和讲座。
3. In work or study, it is necessary to write down creative thinking, work descriptions, reports, papers, etc. in English.在实际工作或学习中，需要用英语撰写创作思路、作品说明、报告或论文等。
4. It is necessary to engage in oral communication or presentations related to art profession in English, or to present papers in English at academic conferences.需要用英语进行与专业相关的口头交流或陈述，或在学术研讨会上用英语宣读论文等。
5. There is a demand for study tours, further education abroad, or visiting scholars. 有游学、留学深造或访学的需求。
6. Students are not accustomed to listening to lectures by foreign experts or teachers, and cannot keep up with the pace and do not know how to take notes.学生不习惯听外国专家或教师的讲座，跟不上，不会笔记。（教师请根据自身观察选择；学生请根据自身经历选择）
7. Students read original textbooks and professional literature at a slow speed.学生阅读原版教材和专业文献速度慢。（教师请根据自身观察选择；学生请根据自身经历选择）
8. Students have difficulties in using English for professional oral communication and participating in academic discussions. 学生用英语做专业口头交流、参加学术讨论有困难。（教师请根据自身观察选择；学生请根据自身经历选择）
9. Students have difficulties in writing work description, literature review, abstract, papers, etc..学生用英语撰写作品介绍、文献综述、论文摘要、小论文等有困难。（教师请根据自身观察选择；学生请根据自身经历选择）
10. Students lack the methods and necessary knowledge to read original textbooks or works.学生缺少阅读艺术原版教材或著作的方法及必要知识储备。（教师请根据自身观察选择；学生请根据自身经历选择）
11. ESP can improve students’ international communication and competitiveness in their professional fields.专门用途英语能提高学生在专业领域内的国际交往和国际竞争能力。
12. ESP can enhance students’ competitiveness in further studies or future employment.专门用途英语能提高学生今后学习上继续深造或工作就业中的竞争力。
13. Teaching ESP is more effective than teaching general English in improving students’ language proficiency and skills.专门用途英语教学比通用英语教学在提高学生英语水平和能力方面更有效。
14. Teaching ESP is more effective than teaching general English in motivating students to learn the language.专门用途英语教学比通用英语教学在调动学生英语学习积极性方面更有效。
15. University students must have a solid foundation in general English, that is, they need to learn general English well before studying ESP.大学生必须有扎实的英语基础，即学好通用英语才能进入专门用途英语学习。
16. As long as university students learn general English well and have a solid foundation, they can meet the requirements of using ESP without studying it specifically.大学生只要学好通用英语，基础打扎实了，不学习专门用途英语也可以胜任专业英语的运用需求。
17. College English is a course for general education, and more courses on English culture, literature, and other general knowledge should be offered. 大学英语是一门素质教育课程，应多开设英美文化、英美文学等通识类课程。
18. College English is a practical course, and more ESP courses should be offered.大学英语是一门工具性课程，应多开设专门用途英语类课程。
19. The lack of qualified teachers is one of the main problems in conducting specialized English teaching. 缺少合格的教师是开展专门用途英语教学的主要问题之一。
20. The lack of good specialized English textbooks is one of the main problems in conducting specialized English teaching. 缺少好的专门用途英语教材是开展专门用途英语教学的主要问题之一。
21. The inability to change mindset is one of the main problems in conducting specialized English teaching. 观念不能转变是开展专门用途英语教学的主要问题之一。
22. The low English proficiency of students is one of the main problems in conducting specialized English teaching.学生的英语水平低是开展专门用途英语教学的主要问题之一。
