# Supplementary material for: A needs analysis of ESP courses in colleges of art and design: Consensus and divergence
Source: PLoS One. 2024 Jun 11;19(6):e0305210. doi: 10.1371/journal.pone.0305210 (PMC11166353; doi:10.1371/journal.pone.0305210)
Supplement: Attachment — Submitted filename: Response to Reviewers.docx [file pone.0305210.s003.docx]

**Subject:** Response to Reviewer Comments on Manuscript PONE-D-23-32993, Entitled "A Needs Analysis of ESP Courses in Colleges of Art and Design: Consensus and Divergence"

Dear editors and reviewers,

I am writing to submit the revised version of my manuscript referenced above. I would like to express my gratitude to you for the insightful comments and suggestions. I have carefully considered each point raised and have made significant revisions to the manuscript accordingly. Below, I detail how each comment was addressed:

**Reviewer's Comment: Descriptive Literature Review**

**Revision Made:** I have enriched the Literature Review section by incorporating more critical commentaries, thereby enhancing its critical depth and academic rigor. This addition aims to provide a more analytical perspective on existing literature.

**Reviewer's Comment: Lack of Theoretical Framework**

**Revision Made:** A theoretical framework has been developed and included, based on the insights gained from the revised literature review and the research design. This framework provides a stronger academic foundation for the study.

**Reviewer's Comment: Descriptive Data Analysis**

**Revision Made:** The data analysis section has been thoroughly revised. The focus now is on interpreting the implications and potential reasons behind the observed statistical trends and differences, moving beyond mere description to more inferential analysis.

**Reviewer's Comment: Missing Critical Discussion**

**Revision Made:** A comprehensive discussion section has been added. This section ties directly to the study’s title and is based on the updated data analysis, offering a critical examination of the findings in the context of existing literature.

**Reviewer's Comment: Absence of Pedagogical Implications in Conclusion**

**Revision Made:** The Conclusion and Implications section has been rewritten to explicitly include pedagogical implications. This addition is intended to highlight the practical relevance of the study’s findings in educational contexts.

Additionally, I have made the following improvements:

**Introduction:** The introduction has been revised for enhanced clarity and coherence, setting a clear context for the study.

**Supporting Information:** The questionnaire used in the research, along with the original collected data, has been included in the Supporting Information part, providing a comprehensive view of the research methodology.

I believe these revisions address the concerns raised by the reviewers and enhance the manuscript's contribution to the field. I hope that the revised manuscript is now suitable for publication in PLOS ONE.

Thank you for considering my submission, and I look forward to hearing from you.

Sincerely,

Fengfan Mao

Hubei Institute of Fine Arts
