# Supplementary material for: A needs analysis of ESP courses in colleges of art and design: Consensus and divergence
Source: PLoS One. 2024 Jun 11;19(6):e0305210. doi: 10.1371/journal.pone.0305210 (PMC11166353; doi:10.1371/journal.pone.0305210)
Supplement: Attachment — Submitted filename: Response to Reviewers.docx [file pone.0305210.s005.docx]

Dear Dr. Derakhshan,

Thank you for your valuable feedback on my manuscript titled “A Needs Analysis of ESP Courses in Colleges of Art and Design: Consensus and Divergence”. I have carefully considered your comments and made substantial revisions to improve the quality of the paper. Below is a summary of the changes made in response to your suggestions:

**Literature Review:** I have restructured and rewritten the literature review section with a new structure and perspective. The revised version includes more critical comments and a thorough analysis of the existing literature to highlight the gaps and the relevance of my study.

**Theoretical Framework:** Based on the revised literature review, I have enriched the theoretical framework. This section now clearly outlines the theories and models that underpin the research.

**Data Analysis:** The data analysis section has been reformulated from a new perspective to include inferential statistics. I have incorporated a more robust analysis of the data, moving beyond descriptive statistics to offer deeper insights. Moreover, the formatting of tables in this part has been enhanced.

**Discussion:** The discussion section has been revised to reflect the new data analysis. It now includes a critical discussion of the findings, their implications, and how they relate to the existing literature.

**Pedagogical Implications:** In the conclusion section, I have added the pedagogical implications of my findings. This part highlights how the results can be applied in educational settings and suggests practical recommendations for educators.

Additionally, I have made the following improvements:

**Abstract:** The abstract part has been improved to respond to the changes in other parts of the paper.

**References：**As the paper undergoes revisions, additional references are included in the reference list.

I believe these revisions have significantly strengthened the manuscript, and I look forward to your feedback.

Thank you for your guidance and consideration.

Best regards,

Fengfan Mao

Hubei Institute of Fine Arts
